# Multiomic sequencing of paired primary and metastatic small bowel carcinoids

**DOI:** 10.12688/f1000research.130251.1

**Published:** 2023-04-18

**Authors:** Mackenzie D. Postel, Sourat Darabi, James R. Howe, Winnie S. Liang, David W. Craig, Michael J. Demeure

**Affiliations:** 1 Institute of Translational Genomics, Keck School of Medicine of USC, Los Angeles, CA, USA; 2 Precision Medicine, Hoag Family Cancer Institute, Newport Beach, CA, 92663, USA; 3 Department of Surgery, University of Iowa Carver College of Medicine, Iowa City, IA, USA; 4 Translational Genomics Research Institute, Phoenix, AZ, USA

**Keywords:** Carcinoids, whole exome sequencing, Whole transcriptome, Splicing variants, Small bowel carcinoids, Metastatic carcinoids

## Abstract

**Background:** Small bowel carcinoids are insidious tumors that are often metastatic when diagnosed. Limited mutation landscape studies of carcinoids indicate that these tumors have a relatively low mutational burden. The development of targeted therapies will depend upon the identification of mutations that drive the pathogenesis and metastasis of carcinoid tumors.

**Methods:** Whole exome and RNA sequencing of 5 matched sets of normal tissue, primary small intestine carcinoid tumors, and liver metastases were investigated. Germline and somatic variants included: single nucleotide variants (SNVs), insertions/deletions (indels), structural variants, and copy number alterations (CNAs). The functional impact of mutations was predicted using Ensembl Variant Effect Predictor.

**Results:** Large-scale CNAs were observed including the loss of chromosome 18 in all 5 metastases and 3/5 primary tumors. Certain somatic SNVs were metastasis-specific; including mutations in *ATRX*, *CDKN1B*, *MXRA5* (leading to the activation of a cryptic splice site and loss of mRNA), *SMARCA2*, and the loss of *UBE4B.* Additional mutations in *ATRX*, and splice site loss of *PYGL*, leading to intron retention observed in primary and metastatic tumors.

**Conclusions:** We observed novel mutations in primary/metastatic carcinoid tumor pairs, and some have been observed in other types of neuroendocrine tumors. We confirmed a previously observed loss of chromosome 18 and *CDKN1B.* Transcriptome sequencing added relevant information that would not have been appreciated with DNA sequencing alone. The detection of several splicing mutations on the DNA level and their consequences at the RNA level suggests that RNA splicing aberrations may be an important mechanism underlying carcinoid tumors.

## Introduction

Small intestine neuroendocrine tumors (SI-NETs), or carcinoid tumors, are the most common malignancy of the small bowel ( Bilimoria et al. 2009). These tumors are characterized by their secretion of biogenic amines (such as serotonin and histamine), peptides (such as somatostatin and chromogranins), tachykinins, and/or prostaglandins ( Pinchot et al. 2008; de Mestier et al. 2021). Secretion of these substances can lead to carcinoid syndrome, which is characterized by diarrhea, abdominal pain, bronchoconstriction, skin flushing, and valvular heart disease ( Eltawil et al. 2022; Tran, Sherman, and Howe 2021; Moertel 1987).

It has been postulated that carcinoid tumors are derived from enterochromaffin cells within intestinal crypts ( Pinchot et al. 2008). Anatomically, small bowel carcinoids most frequently occur in the terminal ileum ( Keck et al. 2018). Although these tumors tend to be indolent and insidious, they are often already metastatic at the time of diagnosis ( Yao et al. 2008; Shah et al. 2019). The median age of diagnosis for small bowel carcinoid tumors is 61 years ( Shah et al. 2019). Carcinoids are generally well-differentiated and slow-growing ( Cunningham et al. 2011; 'StatPearls' 2022). These tumors most often spread to regional lymph nodes, adjacent mesentery, and the liver ( Pinchot et al. 2008). Improved imaging modalities, including the intravenous use of radiopharmaceutical gallium ^68^Ga-or copper ^64^Cu-dotatate paired with positron emission tomography (PET), may demonstrate metastases that are not appreciated with other, less sensitive imaging studies ( Sanli et al. 2018).

Surgical removal of a localized, primary carcinoids (along with adjacent mesentery and lymph nodes) can be curative ( Quinones et al. 2022). Treatment of metastatic carcinoid tumors, however, requires selection from a repertoire of therapeutic options, including surgical resection, administration of somatostatin analogues, peptide receptor radiotherapy with ^177^Lu-dotatate, hepatic arterial embolization, hepatic radiofrequency ablation, external beam radiotherapy of selected isolated metastases, and administration of targeted therapies ( Howe et al. 2017).

As the incidence of carcinoid tumors is increasing at an annual rate of 6.3%, the development and adoption of more effective targeted therapies is imperative ( Ahmed 2020; Dasari et al. 2017; Maggard, O'Connell, and Ko 2004). Better elucidation of molecular drivers of carcinogenesis and metastasis underlying carcinoid tumors will help foster the development of new treatments. Knowledge of the full genomic landscape of carcinoid tumors is limited, as prior studies have deemed carcinoids to be relatively “mutationally silent” compared to other malignancies ( Lim and Pommier 2021). Loss of chromosome 18 as well as various loss of function mutations in cyclin-dependent kinase inhibitor 1B ( *CDKN1B*, which encodes the cell cycle regulatory protein p27) have been reported in a *minority* of patients (i.e. 9%) ( Crona et al. 2015; Crona and Skogseid 2016; Cunningham et al. 2011; Francis et al. 2013; Kulke et al. 2008; Kytölä et al. 2001; Lim and Pommier 2021; Löllgen et al. 2001; Banck et al. 2013).

Reported aberrations include germline mutations in *CDKN1B,* which are known to cause Multiple Endocrine Neoplasia type IV (MEN4) ( Seabrook et al. 2022). It has been suggested that loss of chromosome 18q may be an early event in the evolution of carcinoids, whereas loss of *CDKN1B* and, therein, loss of tumor suppressor p27, occur later in malignant progression ( Crona and Skogseid 2016; Di Domenico et al. 2017).

Recent studies have demonstrated that analysis of RNA may reveal the presence of causative molecular drivers of disease when DNA-level analysis has failed to do so ( McCullough et al. 2020). Therefore, the integration of genomic data with transcriptomic data is the next logical step toward characterizing the molecular alterations underlying carcinoid tumors and, in doing so, identifying biomarkers for precision diagnosis, prognosis, and treatment. In this study, we investigated the genomic and transcriptomic landscapes of trio sets of germline, primary tumor, and metastatic tumor samples derived from 5 patients. We observed consistent loss of chromosome 18, as well as loss of function (LOF) mutations in *CDKN1B.* We identified metastasis-specific mutations in primary and metastatic carcinoid tumor pairs, several of which have been reported as driver mutations in *other* neuroendocrine tumor types. Transcriptome sequencing added relevant information that would not have been appreciated from DNA data alone. The detection of several splicing mutations on the DNA level, and of their consequences at the RNA level, suggest that RNA splicing aberrations may be an important mechanism underlying the development of carcinoid tumors.

## Methods

### Samples

Five patients (here referred to as 0006, 0007, 0008, 0009, 0018) undergoing surgical resection of their carcinoid tumors at Hoag Hospital (Newport Beach CA) or the University of Iowa Neuroendocrine Tumor Clinic consented to this study (written consent), which received IRB approval from the participating institutions (Hoag IRB number: 20180303; Iowa IRB number: 199911057). These patients had tissue available from normal tissue, primary tumors, and liver metastases. Deidentified tissue samples were sent for DNA/RNA extraction and sequencing as detailed below at the Translational Genomics Research Institute (TGen) (Phoenix AZ).

### Sequencing

Tumor or constitutional DNA was extracted using the Qiagen AllPrep Kit or GeneRead FFPE DNA Kit (Cat. No. 80234, 180134), and tumor RNA was extracted using the Qiagen AllPrep Kit or RNeasy Mini Kit (Cat. No. 74104). Using 200 ng of input DNA, whole exomes were constructed for each sample using the Kapa Hyper Prep Kit (Cat. No. 07962363001) using Agilent SureSelect Human All Exon V7 baits. RNA libraries were constructed using 500ng of input RNA per sample and using the Illumina TruSeq Stranded Total RNA Kit with Ribo-zero (Cat. No. RS-122-2201). Paired-end sequencing of libraries was performed on the Illumina NovaSeq 6000 using S1 and SP flowcells for 100bp reads. Approximate sequencing targets were 200× for tumor exomes, 100× for constitutional exomes, and 150 million total reads for each RNA library.

### Bioinformatics

Paired DNA and RNA data (for germline, primary, and metastatic tissue samples) were input into two pipelines: TGen’s Phoenix pipeline, and the Keck School of Medicine of USC’s Genomics Platform (KGP) “Echo” pipeline.

### TGen Phoenix pipeline

This pipeline utilizes *SAMtools* (v1.10) and *Burrows-Wheeler Aligner* ( *bwa* v0.7.17) for alignment of whole exome sequencing (WES) data; *STAR* (v2.7.5a) for RNA sequencing (RNAseq) alignment; *DeepVariant* (0.10.0-gpu) to call germline SNVs and small indels; *lancet* (v1.1.x) for somatic variant calling; *Manta* (v1.6) for detection of somatic structural variants and indels; *Octopus* (v0.6.3-beta) for haplotype-based variant calling; *Strelka2* (v2.9.10) to call small somatic variants; *Vardict* ( *Java* v1.7.0), which calls somatic SNVs, multi-nucleotide variants, indels, structural variants, and loss of heterozygosity; SnpEff (v4.3T) for SNP annotations; and *vcfMerger2* (v0.8.7).

### USC KGP Echo pipeline

Whole exome FASTQs (tumor/normal pairs, for both primary and metastatic tumors) were aligned to human genome build GRCh38 (Gencode v29 primary assembly) using *bwa mem* (v0.7.17). Base quality score recalibration was performed using *GATK’s BaseRecalibrator* (v4.0.10.1) and *ApplyBQSR.* SAM files were merged, and duplicate reads were marked using *GATK’s MergeSamFiles* and *MarkDuplicates*, respectively. Quality control metrics for the resulting sorted, indexed BAMs were retrieved using *Samtools Stats*, *Picard HSMetrics*, *Picard GCBias Metrics*, and *Picard MultiMetrics.* Variants and mutations were called using a *dbSNP* reference variant call format (VCF) file (v146 hg38) and *GATK HaplotypeCaller.* Resulting VCFs were annotated with *GATK’s Mutect2* (for detection of somatic point mutations), *Manta* (v1.5.0, for detection of structural variants and indels), *Strelka* (v2.9.0, for detection of somatic SNVs and small indels), TGen’s *Seurat* (v2.5, for detection of somatic point mutations), TGen’s *tCoNuT* (v1.0, for copy number analysis), TGen’s translocation tool, *Freebayes* (v1.2.0, a Bayesian small SNV/indel detector), and *SnpEff* (predictor of variant effects). The final variant candidate set was annotated with *Ensembl* Variant Effect Predictor ( *VEP*).

RNAseq FASTQs (tumors only) were aligned to human genome build GRCh38 using *STAR* (v2.6.1d). Duplicates were marked using *GATK’s MarkDuplicates.* Gene fusion predictions were made using *STAR-Fusion* (v2.6.1d). Quality control metrics for the resulting sorted, indexed BAMs were retrieved using *Samtools Stats*, *Picard RNA Metrics*, and *Picard MultiMetrics.* Variants and mutations were called using a *dbSNP* reference VCF (v146 hg38) and *GATK’s RNA HaplotypeCaller.* *Salmon*, *featureCounts*, *HTSeq Counts*, and *Cufflinks* were used for gene quantification. The final variant candidate set was annotated with *VEP.* We utilized *MultiQC* to compile analysis logs and create a comprehensive quality control report. Differential expression and pathway analyses were run using *iDEP* with *GAGE* analysis, *KEGG* gene sets, and *Pathview* for visualization ( Ge, Son, and Yao 2018; Luo and Brouwer 2013; Kanehisa et al. 2021).

## Results

### Bioinformatic summary statistics

Tumor samples had, on average, a 45-fold gene enrichment, a coverage ≥30× for 95% of target bases, 87% aligned reads, and 96% RNAseq correct strand mapping. Constitutional samples had, on average, a 43-fold gene enrichment, a coverage of ≥30× for 91% of target bases, and 99.8% aligned reads.

### Patient-specific molecular findings

Patient 0006 is a 66-year-old male with grade 1 disease, treated with Octreotide ( Table 1). He had large-scale copy number loss of chromosome 18 ( Table 2). He also had a PYGL mutation (rs74464749) in both his primary and metastatic tumors ( Table 3). He had a metastasis-specific mutation in MXRA5 (chrX:3,317,443:G:C).

**Table 1.** Carcinoid patient demographics and sample information.

Patient ID	Age	Sex	Stage	Grade	Treatment	Outcome
**0006**	66	M	4	1	Octreotide	Alive
**0007**	62	F	4	2	Lutetium Lu 177 Dotatate	Deceased
**0008**	67	M	4	1	Surgery, Everolimus, Temozolomide	Deceased
**0009**	52	F	4	2	Surgery	Alive
**0018**	71	F	4	2	Surgery, Lanreotide	Alive

**Table 2.** Copy number alterations in primary and metastatic tumor pairs.

Patient ID	Tumor	Copy Number Alterations										
		Chr18 Loss	Chr5 Gain	Chr7 Gain	Chr10 Gain	Chr9 Loss	Chr11q Loss	Chr14q Gain	Chr15q Gain	Chr20 Gain	Chr1 Loss	Chr16q Loss
**0006**	Primary											
	Metastasis	✓										
**0007**	Primary	✓	✓	✓	✓		✓	✓	✓	✓		
	Metastasis	✓	✓	✓	✓		✓	✓	✓	✓		
**0008**	Primary		✓								✓	
	Metastasis	✓	✓	✓	✓	✓						
**0009**	Primary	✓										
	Metastasis	✓										
**0018**	Primary	✓										
	Metastasis	✓										✓

**Table 3.** Point mutations in primary and metastatic tumor pairs – highlighting those that are metastasis-specific.

Patient ID	Tumor	Point Mutations						
		ATRX (X:77683393:G:T)	ATRX (X:77684450:T:A)	CDKN1B (rs797044482)	MXRA5 (X:3317443:G:C)	PYGL (rs74464749)	SMARCA2 (rs752254994)	UBE4B (1:10105515:G:A)
**0006**	Primary					✓		
	Metastasis				✓	✓		
**0007**	Primary							
	Metastasis							
**0008**	Primary							
	Metastasis			✓				✓
**0009**	Primary	✓						
	Metastasis	✓					✓	
**0018**	Primary							
	Metastasis		✓					
**Metastasis-Specific?**		*No*	**Yes**	**Yes**	**Yes**	*No*	**Yes**	**Yes**

Patient 0007 was a 62-year-old female with grade 2 disease, treated with ^177^Lu-dotatate ( Table 1). She succumbed to her disease. She had large-scale copy number changes in both her primary and metastatic tumors; these included: gain of chromosomes 5, 7, 10, 14q, 15q, and 20, and loss of chromosomes 18 and 11q ( Table 2). She did not have any apparent pathogenic point mutations.

Patient 0008 was a 67-year-old male with grade 1 disease, treated with surgical resection, everolimus, then temozolomide ( Table 1). He succumbed to his disease. He had one copy number alteration observed in both his primary and metastatic tumors, gain of chromosome 5 ( Table 2). He also had metastasis-specific copy number changes including gain of chromosomes 7 and 10, and loss of chromosomes 9 and 18. He had metastasis-specific CDKN1B (rs797044482) and a UBE4B (chr1:10,105,515:G:A) mutations ( Table 3).

Patient 0009 is a 52-year-old female with grade 2 disease, treated by surgical resection ( Table 1). The only copy number alteration observed in her tumors was loss of chromosome 18 in the primary/metastatic pair ( Table 2). An ATRX point mutation (chrX:77,683,393:G:T) was identified in both her primary and metastatic tumors resulting in loss of heterozygosity (LOH) and allele-specific expression (ASE) in the mRNA (monoallelic expression of the wild-type allele in mRNA) ( Table 3). She had a metastasis-specific mutation in SMARCA2 (rs752254994).

Patient 0018 is a 71-year-old female with grade 2 disease, treated with surgical resection then monthly lanreotide ( Table 1). She had copy number loss of chromosome 18 in both her primary and metastatic tumors ( Table 2). She had metastasis-specific, large-scale copy number loss of chromosome 16q. She had a metastasis-specific ATRX mutation (chrX:77,684,450:T:A) with monoallelic expression of the wild-type allele in mRNA ( Table 3).

### Differential expression & pathway analyses

Using iDEP’s GAGE analysis tool with KEGG gene sets, it was determined that the top 20 pathways with differentially expressed genes between metastatic vs. primary carcinoids included: “pathways in cancer,” “chemical carcinogenesis,” and “viral carcinogenesis” ( Figure 5; Tables 4 & 5). The pathways within the “pathways in cancer” framework that appear to have the most significant changes in expression between metastases vs. primary tumors include: cytokine-cytokine receptor interaction, p53 signaling, extracellular membrane receptor and focal adhesion interactions, Wnt signaling, PI3K-Akt signaling, MAPK signaling, calcium signaling, TGF-b signaling, HIF-1 signaling, Notch and Hedgehog signaling, estrogen and androgen signaling, cell cycle, and block of differentiation ( Figure 5).

**Table 4.** Top differentially expressed genes.

Regulation	Ensembl ID	log2 Fold Change	Adj.Pval	Symbol	Chr	Type	#0006_Primary	#0006_Met	#0007_Primary	#0007_Met	#0008_Primary	#0008_Met	#0009_Primary	#0009_Met	#0018_Primary	#0018_Met
Up	ENSG00000249631	5.004112931	2.98E-03		4p15.33	IncRNA	2.808146768	5.910458486	2.256831517	4.787822064	2.208600152	6.275193112	2.152317068	3.849774557	2	3.410960355
Up	ENSG00000255693	4.988902949	8.03E-02	LINC02389	12q14.3	IncRNA	3.000923778	4.892153345	2.256831517	2.478945215	2.390813802	7.749324524	2.638550945	2.246142115	2	5.748843171
Up	ENSG00000251152	4.523601477	9.34E-02		4p15.33	IncRNA	3.000923778	7.492326457	2.474776918	3.982923261	2.208600152	7.411787314	2.919014773	4.498450824	3.651237056	2
Up	ENSG00000200834	2.737814169	6.00E-02	Y_RNA	9p13.3	misc_RNA	2.585578419	5.066166275	2.664077712	3.470802033	2.95113892	4.932045885	2.831448134	4.944320187	3.651237056	4.767017838
Up	ENSG00000260955	2.274889033	6.62E-02		8q11.23	IncRNA	3.323036558	5.221431391	3.241206602	3.90922402	3.062788147	5.025200891	3.079654245	5.012423927	3.049989149	4.475769259
Up	ENSG00000242986	1.848200194	1.31E-02	RPL21P99	12p11.22	processed_pseudogene	5.359218883	6.041299761	4.733505776	6.115995297	4.616755478	6.72810925	4.35616047	6.133957023	4.667340679	7.077586434
Down	ENSG00000115850	-11.00672978	6.29E-03	LCT	2q21.3	protein_coding	13.67912559	2	2.664077712	2	9.747547114	2	12.66313063	3.607079139	2	2.50058312
Down	ENSG00000114113	-10.85396153	4.48E-02	RBP2	3q23	protein_coding	10.21372903	2	2	2	8.065230864	2	12.79664791	2.45634636	2	2.871505065
Down	ENSG00000148942	-9.950915626	2.87E-05	SLC5A12	11p14.2	protein_coding	9.710921957	2.56153175	2.256831517	2.259257005	7.718307982	2	11.32681209	2.246142115	2	2
Down	ENSG00000137860	-9.285099263	1.16E-04	SLC28A2 ENSG00000137860	15q21.1	protein_coding	6.586732557	2	4.108517157	2.259257005	6.102566161	2.327727231	10.10583006	2	2	2
Down	ENSG00000110244	-8.522081459	2.73E-02	APOA4	11q23.3	protein_coding	14.5943037	2	9.407195823	2	11.68858384	6.004235855	14.21106546	5.828654339	2	4.363970817
Down	ENSG00000204978	-8.482267717	6.29E-03	ERICH4	19q13.2	protein_coding	5.932463263	2	2.256831517	2	7.3785928	2	8.689606784	2.246142115	2	2
Down	ENSG00000081800	-8.44153797	8.14E-03	SLC13A1	7q32.32	protein_coding	6.783139558	2	2	2.259257005	5.715254076	2	9.087036065	2.246142115	2	2
Down	ENSG00000237070	-7.253199904	3.18E-02		7p21.2	IncRNA	3.323036558	2.307915358	5.643423062	2	2.552573002	2	7.280214099	2	2	2
Down	ENSG00000172689	-6.943021898	1.29E-02	MS4A10	11q12.2	protein_coding	8.753373894	3.279474842	2	2.988637996	6.703000403	2	9.916678007	2.246142115	2	2.50058312
Down	ENSG00000257335	-6.905894979	2.87E-05	MGAM	7q34	protein_coding	11.69924675	3.130659634	5.928291766	4.51719801	9.684575926	5.933755997	13.38815903	4.7182936118	5.086376154	4.676314515
Down	ENSG00000141434	-6.864967636	8.94E-04	MEP1B	18q12.1	protein_coding	10.90873372	4.181804211	5.55051177	6.356802554	10.80265254	4.832045885	13.56049296	3.849774557	2	3.620082636
Down	ENSG00000224057	-6.704746578	9.34E-02	EGFR-AS1	7p11.2	IncRNA	4.088875439	2	2.664077712	2.259257005	3.263093034	2	6.941633957	2	2	2
Down	ENSG00000108576	-6.373802454	1.13E-02	SLC6A4 ENSG00000108576	17q11.2	protein_coding	8.862917994	2.307915358	2.831396233	3.831556257	6.737062329	2	10.38491054	3.607079139	2	2.871505065
Down	ENSG00000196549	-6.368097476	7.75E-05	MME	3q25.2	protein_coding	11.98234177	5.458453109	8.657524291	6.575070858	9.713734749	4.483009385	13.37581921	3.773324275	3.651237056	5.216564396
Down	ENSG00000107165	-6.295095692	2.98E-03	TYRP1	9p23	protein_coding	5.955924188	2.307915358	2.831396233	3.470802033	8.824245143	3.014454367	8.827537465	2	4.074348836	2
Down	ENSG00000181778	-6.264410935	5.51E-02	TMEM252	9q21.11	protein_coding	4.80893818	2	6.342719293	3.662425974	5.027871338	2	9.283371766	2.246142115	2	2
Down	ENSG00000130234	-6.069239584	2.00E-03	ACE2	Xp22.2	protein_coding	7.305580984	2.56153175	3.355474865	3.125104159	4.91337553	2.594614818	9.392570078	2.948717924	3.049989149	2
Down	ENSG00000182156	-5.981823698	1.00E-03	ENPP7	17q25.3	protein_coding	9.249762336	3.130659634	7.874700848	2	6.68566304	3.477803081	6.022843075	2.639782253	2	2.50058312
Down	ENSG00000241224	-5.801523377	6.62E-02	C3orf85	3q13.13	protein_coding	6.908676034	2	6.954529268	3.831556257	5.9069844	2	9.327047569	2	2	3.410960355
Down	ENSG00000174358	-5.440433188	8.94E-04	SLC6A19	5p15.33	protein_coding	10.74077933	5.257770368	6.328797872	5.38534592	8.178016908	4.483009385	11.35382556	3.515173157	3.651237056	3.802694387
Down	ENSG00000162670	-5.419229547	8.78E-02	BRINP3	1q31.1	protein_coding	4.955805091	2.777159177	8.523494902	4.980057382	3.519439604	2	8.859708081	2	8.865616263	2
Down	ENSG00000204876	-5.372532894	2.73E-02		7q36.3	IncRNA	5.674127533	2.56153175	5.620750157	2.988637996	5.081882725	3.185976506	8.414375443	2	2	2
Down	ENSG00000198074	-5.219515781	2.35E-02	AKR1B10	7q33	protein_coding	9.40057972	3.130659634	7.536066463	5.960493133	7.953778005	5.069703154	11.82862207	2.802503691	2	5.150709207
Down	ENSG00000273777	-5.109157311	9.91E-02	CEACAM20	19q13.31	protein_coding	6.586732557	3.756671754	3.739078989	2.669558802	3.595676995	2.594614818	8.662903422	2.246142115	2	2
Down	ENSG00000196611	-5.10807383	1.00E-03	MMP1	11q22.2	protein_coding	6.024101188	3.414364845	3.652311288	2.988637996	8.708838585	3.477803081	5.775496899	2	7.588043813	2.871505065
Down	ENSG00000135220	-5.103761317	3.93E-03	UGT2A3	4q13.2	protein_coding	9.404848084	5.998990901	9.089869395	5.978622305	7.170022714	3.720415122	12.35247694	4.872841576	3.651237056	5.397811389
Down	ENSG00000179674	-5.054467235	4.87E-02	ARL14	3q25.33	protein_coding	5.493535983	3.414364845	5.259042381	5.149663601	11.61464664	5.383536093	9.315999926	5.465411845	5.086376154	2
Down	ENSG00000144820	-4.996451824	1.69E-02	ADGRG7	3q12.2	protein_coding	7.461236634	2.777159177	8.845342257	4.51719801	5.936601016	3.339256038	10.67240777	3.849774557	2	5.279544245
Down	ENSG00000172782	-4.458108815	6.87E-02	FADS6	17q25.1	protein_coding	5.586655643	2.307915358	6.118321451	2.837901775	3.865771836	3.720415122	7.818997991	2.246142115	2	2.50058312
Down	ENSG00000138823	-4.389308515	6.52E-02	MTTP	4q23	protein_coding	11.64254149	7.37316105	12.45385497	5.084193885	9.197371128	6.755610669	13.49699104	7.564433049	3.049989149	9.031525264
Down	ENSG00000144410	-4.241040862	1.29E-02	CPO	2q33.3	protein_coding	8.667164698	4.981781827	5.476677998	4.980057382	6.976868259	4.547365658	11.00719495	5.920138993	5.898855105	3.802694387
Down	ENSG00000170482	-4.133358603	4.48E-02	SLC23A1	5q31.2	protein_coding	7.716057325	4.584065559	7.165045527	3.125104159	5.412119812	3.014454367	9.271983211	3.849774557	2	4.475769259
Down	ENSG00000119125	-3.80975681	5.14E-02	GDA	9q21.13	protein_coding	9.365970417	5.76655658	10.16119653	7.661329568	8.631721192	6.982042643	12.52397145	5.6042943	5.086376154	7.380208496
Down	ENSG00000166268	-3.659510205	2.89E-02	MYRFL	12q15	protein_coding	6.729697766	4.265041534	6.653152077	4.787822064	6.737062329	3.014454367	9.013096455	3.99149961	3.049989149	3.802694387
Down	ENSG00000123496	-3.634542712	3.18E-02	IL13RA2	Xq23	protein_coding	4.6454078	2.96470961	5.371960191	2.259257005	4.541653148	2.327727231	4.982561565	2.802503691	3.049989149	2

**Table 5.** Top 20 downregulated pathways as determined by GAGE with KEGG gene sets.

GAGE Analysis: Metastasis vs. Primary	Statistic	Genes	adj. Pval
Systemic lupus erythematosus	-6.2936	78	5.60E-07
Neutrophil extracellular trap formation	-5.6873	139	2.80E-06
Ribosome	-5.2609	120	2.70E-05
Drug metabolism	-5.1508	39	6.10E-05
Metabolism of xenobiotics by cytochrome P450	-5.0916	42	6.10E-05
Carbon metabolism	-4.8968	94	6.10E-05
Chemical carcinogenesis	-4.7058	194	8.50E-05
Coronavirus disease	-4.667	176	9.00E-05
Peroxisome	-4.5064	67	2.70E-04
Parkinson disease	-4.4826	224	1.80E-04
Fatty acid degradation	-4.3638	35	5.20E-04
Alcoholism	-4.3521	138	2.80E-04
Biosynthesis of cofactors	-4.3137	126	3.10E-04
Glycolysis/Gluconeogenesis	-4.2444	45	5.20E-04
Viral carcinogenesis	-4.2271	160	4.00E-04
Amyotrophic lateral sclerosis	-4.1743	318	4.00E-04
Necroptosis	-4.1396	115	5.20E-04
Glutathione metabolism	-4.1314	47	6.50E-04
Alzheimer disease	-4.0057	310	6.10E-04
Pathways in cancer	-3.9954	429	6.10E-04

## Discussion

### Mutational landscape of carcinoids

Previous genomic analyses have failed to identify a consistent, putative driver mutation in small bowel neuroendocrine tumors. We endeavored to dive deeper into the molecular landscape of carcinoids by supplementing exome analysis with transcriptomic analysis in paired primary small bowel tumors and liver metastases from the same patients. We were particularly interested in discovering drivers of metastatic potential, as metastatic carcinoids pose the greatest morbidity and mortality risks. We intended to probe RNA for potential culprits that were, perhaps, not immediately evident from DNA analysis alone. RNAseq data enabled us to confirm the consequences of alterations found on the DNA level while also observing phenomena (like intron retention and ASE) that would not otherwise be apparent.

Tumor cells gain oncogenic and lose tumor-suppressive functions via various mechanisms, including point mutations, CNAs, and structural variants. In this study, we observed various CNAs, the most frequent being loss of chromosome 18. Loss of 18q has been previously reported as a potential mechanism of early oncogenesis in carcinoid tumors ( Crona and Skogseid 2016). The long arm of chromosome 18 spans several confirmed and putative tumor suppressors, including Retinoblastoma-Binding Protein 8 ( *RBBP8*), SMAD family members 2 and 4 ( *SMAD2/4*) *,* and deleted in colorectal cancer ( *DCC*) ( Cunningham et al. 2011; Lim and Pommier 2021). Though several studies have suggested that *DCC* may be the key tumor suppressor lost, this has not been rigorously confirmed ( Löllgen et al. 2001; Nieser et al. 2017). Other CNAs – which were less consistent across our samples, but which reflect previously reported evidence – included loss of chromosomes 9p, 11q, and 16q, and gain of chromosome 14 ( Table 2) ( Cunningham et al. 2011).

**Figure 1.**
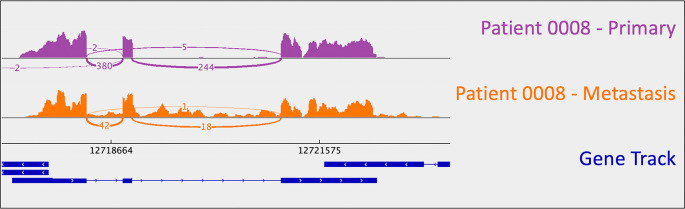
*CDKN1B* sashimi plots for patient 0008's primary/metastatic tumor pair. A loss-of-function frameshift mutation resulting in intron inclusion is observed in the metastatic tumor alone. This is perceptible as the orange signal that reads through intronic regions (i.e., the thin blue lines in the gene track). Sashimi plots were generated using the Broad Institute’s Integrative Genomics Viewer ( *IGV*).

Loss of *CDKN1B* function is another previously reported characteristic of small bowel carcinoid tumors (though it has only ever been reported in a minority, i.e. 9% of tumors in larger studies) ( Crona and Skogseid 2016; Crona et al. 2015; Maxwell et al. 2015). An insertion in *CDKN1B* (rs797044482, reported as “likely pathogenic” for neuroendocrine neoplasms in *ClinVar*) was identified in patient 0008’s metastasis ( Figure 1). Indel presence was confirmed in mRNA. Interestingly, this patient had a “second hit” to *CDKN1B*: uniparental disomy (and resulting LOH). B-allele frequency (BAF) is a measure of allelic balance, whereby a heterozygous SNP would have a BAF of 0.5 and a homozygous SNP – or LOH event – would result in a BAF that deviates from 0.5 (to 0 or 1). Patient 0008’s uniparental disomy event was identified via CNA, as B-allele frequency deviated from 0.5 despite the fact that there was no copy number change at that location. This example exemplifies the ability of cancer cells to “mix and match” mechanisms to gain and lose gene products.

### ATRX mutations as potential drivers

The tumor suppressor gene *ATRX* encodes a chromatin-remodeler that is a member of the SWI-SNF family of proteins; it is involved in transcriptional regulation, DNA recombination, nucleosome remodeling, and DNA repair ( Bradley et al. 2019; Valenzuela et al. 2021). Somatic mutations in this gene have frequently been reported in gliomas, gastro/pancreatic neuroendocrine tumors, pheochromocytomas, and paragangliomas ( Crona and Skogseid 2016; Jiao et al. 2011). *ATRX* has also been found to be mutated in gliomas and acute lymphoblastic leukemia (ALL) ( Bradley et al. 2019). *ATRX* plays an essential role in brain development and is ubiquitously expressed at high levels in brain tissue ( Valenzuela et al. 2021).

Though conflicting evidence exists, it has been reported that *ATRX* escapes X-inactivation, perhaps in a developmental stage and/or tissue-specific manner, with *ATRX* showing biallelic expression in XX-females and monoallelic expression in XY-males ( Valenzuela et al. 2021). *ATRX* deficiency results in impaired nonhomologous end-joining and genomic instability ( Bradley et al. 2019). Notably, *ATRX* mutations are frequently observed in female gastric cancer patients with high microsatellite instability (MSI), tumor mutational burden (TMB), and programmed death-ligand 1 ( *PD-L1*) expression; these characteristics are purported to be predictive biomarkers for immunotherapy response ( Ge et al. 2021).

Two of our patients (0009, 0018) had *ATRX* mutations. Patient 0009, an XX-female, had LOF mutations in *ATRX* in both her primary and metastatic tumors. Patient 0018, another XX-female, harbored a metastasis-specific *ATRX* mutation. Curiously, both mutations were predicted to be “tolerated” by VEP and showed ASE/monoallelic expression of the wild-type allele in RNA.

### Metastasis-specific findings

**Figure 2.**
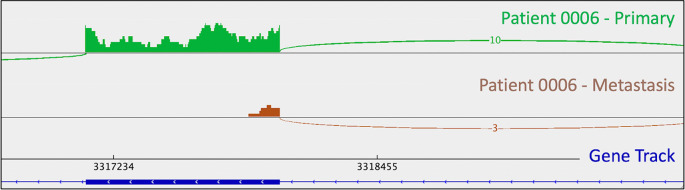
Activation of a metastasis-specific *MXRA5* cryptic splice site in patient 0006 leads to exon truncation. This is appreciable as a lack of mRNA reads corresponding to the downstream portion of the depicted exon (i.e., the thick blue line in the gene track) in the metastatic tumor (brown trace). This phenomenon is not observed in the primary tumor. Sashimi plots were generated in *IGV.*

**Figure 3.**
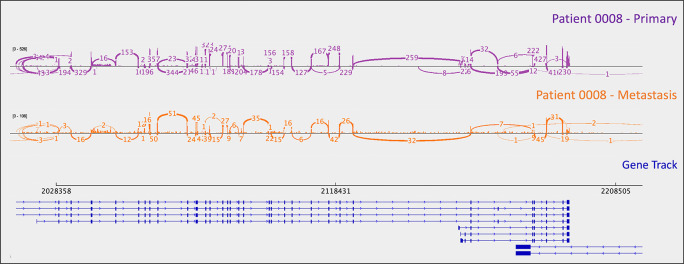
A metastasis-specific *SMARCA2* missense mutation (rs752254994) is observed in the mRNA of patient 0008. Sashimi plots were generated in *IGV.*

**Figure 4.**
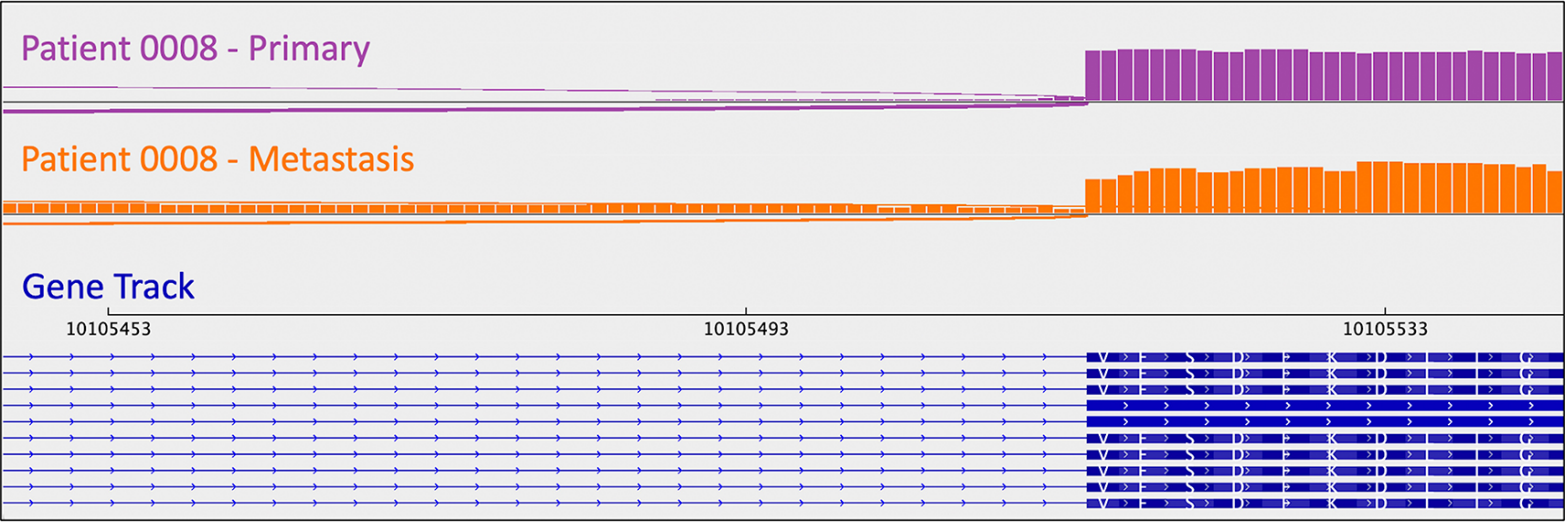
A metastasis-specific *UBE4B* splicing mutation results in intron retention in the mRNA of patient 0008. Intron retention is apparent as the presence of mRNA (i.e., the orange signal/bars) corresponding to intronic material (i.e., the thin blue lines in the gene track). Sashimi plots were generated in *IGV.*

As mentioned above, one metastasis-specific finding was patient 0008’s *CDKN1B* mutation (rs797044482) ( Figure 1). A metastasis-specific missense mutation in the gene Matrix Remodeling Associated 5 ( *MXRA5*, chrX:3,317,443:G:C) was seen in patient 0006 ( Figure 2). *MXRA5* is a purported tumor suppressor gene ( Tegally et al. 2020; Xiong et al. 2012; Cheng et al. 2017). It also plays a role in normal matrix remodeling and anti-inflammatory responses, the disruption of which is essential to metastatic progression ( Poveda et al. 2017). This deleterious *MXRA5* mutation resulted in the activation of a cryptic splice site and loss of mRNA transcripts. As patient 0006 is male, there was no dosage compensation for *MXRA5.* Patient 0006’s primary and metastatic tumors had a splicing mutation in liver glycogen phosphorylase ( *PYGL*, rs74464749), resulting in intron retention and expression of alternative splice variants in mRNA. A metastasis-specific missense mutation in SWI/SNF Related, Matrix Associated, Actin Dependent Regulator of Chromatin, Subfamily A, Member 2 ( *SMARCA2*, rs752254994) was called for patient 0009 (confirmed in mRNA, Figure 3). *SMARCA2* is part of a chromatin-remodeling complex, and mutations in this gene have been reported in neuroendocrine tumors of the lung and thymus ( Fernandez-Cuesta et al. 2014). A metastasis-specific mutation in Ubiquitination Factor E4B ( *UBE4B*, chr1:10105515:G:A) was called for patient 0008, which resulted in intron retention in the mRNA ( Figure 4); this was paired with a “second hit” of large-scale copy loss of chromosome 1. Mutations in *UBE4B* have previously been associated with neuroblastoma, another type of neuroendocrine tumor ( Byron et al. 2016).

**Figure 5.**
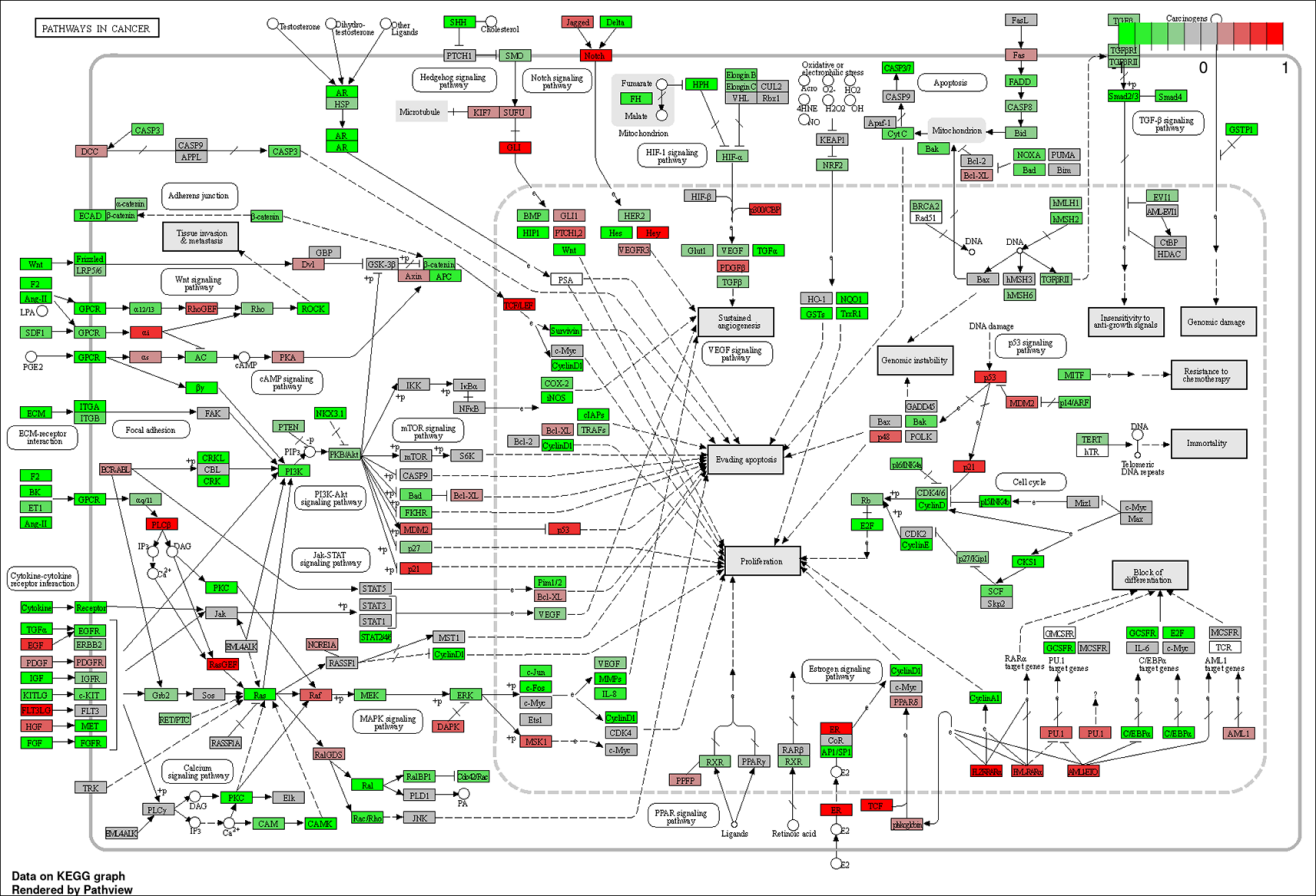
“Pathways in cancer” is one of the top 20 differentially expressed pathways between metastatic and primary carcinoids as determined by *GAGE* Analysis with *KEGG* gene sets. Genes colored bright red are most upregulated, and those colored bright green are most downregulated. The most significant sub-pathways within this framework appear to be: cytokine-cytokine receptor interaction, *p53* signaling, extracellular membrane receptor and focal adhesion interactions, *Wnt* signaling, *PI3K-Akt* signaling, *MAPK* signaling, calcium signaling, cell cycle, *TGF-b* signaling, *HIF-1* signaling, *Notch* and *Hedgehog* signaling, estrogen and androgen signaling, and block of differentiation. These pathways may provide clues regarding which molecular events confer carcinoid tumor metastatic potential.

### RNA splicing results

As mentioned in the section above, patient 0008 had several metastasis-specific splicing mutations illuminated by RNA analysis. This included a mutation in *UBE4B* resulting in intron retention ( Figure 4 *)*, and a mutation in *MXRA5* leading to activation of a cryptic splice site and loss of mRNA.

Splicing mutations were also observed in the tumors of patient 0006. He had a splice-acceptor SNV in *PYGL*, which encodes liver glycogen phosphorylase, in both the primary and metastatic tumor in the liver. The splicing mutation resulted in intron retention. *PYGL* was listed as a mutated gene in carcinoid tumors in a previous study ( Francis et al. 2013).

### Limitations, conclusions, and future directions

Limitations of this study include its relatively small sample size, albeit for a rare tumor type, and the fact that we utilized whole exome data (thereby ignoring introns). It is possible that the molecular drivers of carcinoid tumorigenesis and progression/metastasis are in noncoding regions. Noncoding RNAs – including microRNAs (miRNAs), long noncoding RNAs (lncRNAs), small interfering RNAs (siRNAs), small nuclear RNAs (snRNAs), small nucleolar RNAs (snoRNAs), and PIWI-interacting RNAs (piRNAs) – have been shown to play a role in the pathogenesis of various tumor types ( Byron et al. 2016; Zeuschner, Linxweiler, and Junker 2020). Additionally, given the fact that two of the mutations identified in this study were in genes involved in chromatin remodeling (i.e. *ATRX* and *SMARCA2*), it will be important to study the epigenomic landscape of carcinoid tumors in the future ( Kidd et al. 2021). Lastly – as disparities exist in terms of carcinoid incidence, treatment, and survival – evaluation of the associations between race, ethnicity, and genetic ancestry with carcinoid genetic aberrations may be informative in a larger sample dataset ( Takayanagi et al. 2022; Kessel et al. 2021).

In conclusion, we identified several candidate mutations potentially involved in the pathogenesis and metastatic cascade of carcinoid tumors. Mutations that were identified as metastasis-specific may provide insight into intermediate steps between initial tumorigenesis and metastasis (i.e., drivers of metastatic potential). We confirmed the presence of previously reported molecular aberrations (i.e., loss of chromosome 18 and LOF mutations in *CDKN1B*). It remains unclear whether there is a key tumor suppressor or set of tumor suppressors on chromosome 18, the loss of which is important to the etiology of small bowel carcinoids. Gene knock-outs could be performed to determine which regions on chromosome 18 are necessary and/or sufficient to drive the formation of carcinoid tumors. Additionally, it is worth noting that one potential explanation that could account for the fact that previous studies identified mutations in *CDKN1B* in a minority of carcinoid tumors is the possibility that distinct molecular subtypes of small bowel carcinoids exist. The significant utility of considering transcriptomic data in addition to genomic data was exemplified by our detection of phenomena such as intron retention, splicing variants, and ASE. The addition of RNAseq data also enabled us to confirm the consequences of mutations called on the DNA level in mRNA, and perform differential expression and pathway analyses to identify several pathways potentially involved in conferring metastatic potential to carcinoid tumors.

## Data Availability

European Genome-Phenome Archive: EGAS00001006988, Multiomic Sequencing of Paired Primary and Metastatic Small Bowel Carcinoids, https://ega-archive.org/studies/EGAS00001006988 ( Postel et al. 2023). Further information regarding TGen’s Phoenix and USC’s KGP Echo pipelines can be accessed online at https://github.com/tgen/phoenix and https://kgp.usc.edu, respectively.
