# Supplementary material for: Multiomic sequencing of paired primary and metastatic small bowel carcinoids
Source: F1000Res. 2023 Oct 4;12:417. Originally published 2023 Apr 18. [Version 2] doi: 10.12688/f1000research.130251.2 (PMC10632590; doi:10.12688/f1000research.130251.2)
Supplement: Peer review history

# Reviewer report: Reviewer response for version 2

**DOI:** 10.5256/f1000research.156060.r212102

**Published:** 2023-11-08

**Authors:** Anela Blažević

**Affiliations:** 1 Erasmus MC Cancer Institute, Rotterdam, The Netherlands

The authors addressed the major issues and the text shows improvements making it an interesting and readable article. I fully support indexing the article in its present state.

Is the work clearly and accurately presented and does it cite the current literature?

Partly

If applicable, is the statistical analysis and its interpretation appropriate?

I cannot comment. A qualified statistician is required.

Are all the source data underlying the results available to ensure full reproducibility?

Yes

Is the study design appropriate and is the work technically sound?

Yes

Are the conclusions drawn adequately supported by the results?

Yes

Are sufficient details of methods and analysis provided to allow replication by others?

Partly

Reviewer Expertise:

Neuro-endocrine tumors, proteomics, radiomics, cell culture.

I confirm that I have read this submission and believe that I have an appropriate level of expertise to confirm that it is of an acceptable scientific standard.

# Reviewer report: Reviewer response for version 2

**DOI:** 10.5256/f1000research.156060.r212103

**Published:** 2023-10-19

**Authors:** Phillip Stafford

**Affiliations:** 1 Department of Bioinformatics, Arizona State University, Tempe, Arizona, USA

The authors are quite prolific publishers, I reviewed the literature cited and the revisions.  The statistics support the conclusions, without extending beyond the limitations of their cohort.  The text shows improvements, catching several minor issues, but most importantly the authors directly addressed the major points brought up by the reviewers.  I fully support indexing the article in its present state.

Is the work clearly and accurately presented and does it cite the current literature?

Yes

If applicable, is the statistical analysis and its interpretation appropriate?

Yes

Are all the source data underlying the results available to ensure full reproducibility?

Yes

Is the study design appropriate and is the work technically sound?

Yes

Are the conclusions drawn adequately supported by the results?

Yes

Are sufficient details of methods and analysis provided to allow replication by others?

Yes

Reviewer Expertise:

WTS/WES NGS bioinformatics, cancer biology, immunology.

I confirm that I have read this submission and believe that I have an appropriate level of expertise to confirm that it is of an acceptable scientific standard.

# Reviewer report: Reviewer response for version 1

**DOI:** 10.5256/f1000research.142993.r191552

**Published:** 2023-08-17

**Authors:** Anela Blažević

**Affiliations:** 1 Erasmus MC Cancer Institute, Rotterdam, The Netherlands

Postel et al. investigate small intestinal neuro-endocrine tumors using whole exome and RNA sequencing in a small cohort. Sequencing is done on normal tissue, primary tumor and liver metastases. The study assessed differential expression only on metastatic vs. non-metastatic primary tumors and observed known and novel mutations.

 I do have a few points I would like to have clarified.

 Samples:

- What was the timing between the surgical resection and other treatments as these could affect mutational load. In the result section, patient-specific molecular finding paragraph it is not clearly enough stated.
- Grading. Was the tumor grade determined in the included tissue in the study or on initial biopsy? Was there a discrepancy between the primary tumor and liver metastases?
- What was the origin of the normal tissue? Intestine or healthy liver tissue?
- Was the collection of primary tumor and liver metastases synchronous or metachronous?

Results:

- No mentioning of analysis on normal tissue. If this was deemed not relevant, it should be clarified why the was no differential analysis between normal and primary tumor tissue as this is theoretically also interesting. If there was no significant differential expression, it is also an important observation.
- Table 5 does not add relevant information and the findings are not further discussed, so I would recommend removing it to increase readability and focus on relevant information.

Discussion

- It would be beneficial to discuss the similarities and discrepancies between the finding in this study and published literature in greater details. For example, the statement: Additionally, it is worth noting that one potential explanation that could account for the fact that previous studies identified mutations in CDKN1B in a minority of carcinoid tumors is the possibility that distinct molecular subtypes of small bowel carcinoids exist. What is already know about distinct molecular subtypes? Is there another explanation such as a difference in patient selection or pretreatment? These discrepancy deserve more discussion in order to embed this study properly in the known literature.  
- It would also be insightful to discuss the relation between epigenetic modification and the found RNA splicing results, as small intestinal neuroendocrine tumors are known to have epigenetic dysregulation.

Finally, Postel et. Al. should consider using the term small intestinal neuroendocrine tumor as this is more in line with current guidelines.

 Overall, the study is interesting as it combines whole exome/whole transcriptome NGS and reveals interesting novel data. However, I have significant reservations, as outlines above.

Is the work clearly and accurately presented and does it cite the current literature?

Partly

If applicable, is the statistical analysis and its interpretation appropriate?

I cannot comment. A qualified statistician is required.

Are all the source data underlying the results available to ensure full reproducibility?

Yes

Is the study design appropriate and is the work technically sound?

Yes

Are the conclusions drawn adequately supported by the results?

Yes

Are sufficient details of methods and analysis provided to allow replication by others?

Partly

Reviewer Expertise:

Neuro-endocrine tumors, proteomics, radiomics, cell culture.

I confirm that I have read this submission and believe that I have an appropriate level of expertise to state that I do not consider it to be of an acceptable scientific standard, for reasons outlined above.

# Response

**Published:** 2023-10-04

**Authors:** Mackenzie Postel

**Affiliations:** USC Norris Comprehensive Cancer Center, USA

**REVIEWER** :  “What was the timing between the surgical resection and other treatments as these could affect mutational load. In the result section, patient-specific molecular finding paragraph it is not clearly enough stated.”   

**AUTHOR RESPONSE** : For each patient, surgical resection was the first intervention performed (i.e., sequence was: surgery, medication). Molecular findings, therefore, represent tissues before use of chemotherapeutics/other medications. In the Results section, for each patient, we have used the phrasing: “treated with surgical resection *then* ___” to give a sense of the chronology of treatment.

 —

**REVIEWER** : “Grading. Was the tumor grade determined in the included tissue in the study or on initial biopsy? Was there a discrepancy between the primary tumor and liver metastases?”  

**AUTHOR RESPONSE** : We are happy to clarify. WHO grading by mitotic count and/or Ki-67 labeling index was performed at the time of surgical resection of the tissues used in this study. Primary/met grading was consistent for each pair (no discrepancy between tumors in any given patient). All this to say, in our manuscript, “grade” can be taken to refer to the grade of a given patient’s primary tumor as well as their met. We have endeavoured to make this more clear in the text.

 —

**REVIEWER** : “What was the origin of the normal tissue? Intestine or healthy liver tissue?”

**AUTHOR RESPONSE** : Thank you for your question. The “normal” or constitutional samples were used mainly to differentiate germline from somatic alterations in DNA. We did not have RNA data available for constitutional samples, therefore we did not perform differential expression analysis, eQTLs, or any other transcriptomic comparison between *normal* and tumor (which would, as I believe you are suggesting, require matched healthy liver or intestine). We simply wanted to establish the patients’ germline variants to distinguish them from somatic mutations. Constitutional samples were derived from: gallbladder for patients 0006 and 0007, healthy bowel for patients 0008 and 0009, and peripheral blood for patient 0018. We have added a statement to the Methods section clarifying the sources of constitutional samples.

 —

**REVIEWER** : “Was the collection of primary tumor and liver metastases synchronous or metachronous?”

**AUTHOR RESPONSE** : Thank you for your comment. If you mean metachronous as in two sites with independent primary malignancies, then no — the liver tumors represented metastases from primary small bowel tumors. If you are asking whether primary/metastatic tumors were collected at the same time vs. in sequential surgeries, they were collected at the same time (patients were metastatic at time of presentation/there was no lag time in terms of diagnosis of primary and met as they were diagnosed simultaneously). In this study, one primary tumor and one metastatic tumor were analyzed for each patient (no multiple, synchronous primary tumors for a given patient). I hope this answers your question.

 —

**REVIEWER** : “No mentioning of analysis on normal tissue. If this was deemed not relevant, it should be clarified why the was no differential analysis between normal and primary tumor tissue as this is theoretically also interesting. If there was no significant differential expression, it is also an important observation.”

**AUTHOR RESPONSE**: Thank you for your comment. We did not have RNAseq data available for normal tissue. Should we have the opportunity to gather constitutional transcriptomic data for these patients in the future, it would certainly allow us to investigate the interesting comparison you suggest. We have clarified in the Methods section that differential expression analysis focused on primary/met tumor pairs (and excluded normal tissue) because we only had RNA for tumors.

 —

**REVIEWER**: “Table 5 does not add relevant information and the findings are not further discussed, so I would recommend removing it to increase readability and focus on relevant information.”

**AUTHOR RESPONSE**: Table 5 has been removed per the reviewer’s helpful suggestion.

 —

**REVIEWER**: “It would be beneficial to discuss the similarities and discrepancies between the finding in this study and published literature in greater details.“

**AUTHOR RESPONSE**: Thank you for this consideration. This manuscript was submitted several months ago but had not been assigned reviewers until relatively recently. In this time gap, more multi-omicliterature has been published re: SI-NETs. We have incorporated several of these new studies, including a recent paper by the reviewer.

 —

**REVIEWER**: “It would also be insightful to discuss the relation between epigenetic modification and the found RNA splicing results, as small intestinal neuroendocrine tumors are known to have epigenetic dysregulation.” 

**AUTHOR RESPONSE**: This would be a fascinating future direction. However, currently, we do not have epigenetic data available for these patients.

 —

**REVIEWER**: “Finally, Postel et. Al. should consider using the term small intestinal neuroendocrine tumor as this is more in line with current guidelines.”

**AUTHOR RESPONSE**: Thank you for this feedback. The first sentence of our Introduction section touches on this (defines small bowel “carcinoids” as small intestine neuroendocrine tumors/SI-NETs). We have updated our text to more frequently use the latest terminology “SI-NETs.” We also added this terminology to the manuscript’s “keywords” list.

 —

 Note: we also cited 11 new papers, related to reviewers’ suggestions, 3 of which were published after we had written our original manuscript.

# Reviewer report: Reviewer response for version 1

**DOI:** 10.5256/f1000research.142993.r178411

**Published:** 2023-07-10

**Authors:** Phillip Stafford

**Affiliations:** 1 Department of Bioinformatics, Arizona State University, Tempe, Arizona, USA

Postel et al. investigate small bowel carcinoids using whole exome/whole transcriptome NGS. The methods the group used to asses metastatic vs. non-metastatic conditions are typical of an NGS study of this kind, but with the benefit of using WES/WTS. The cohort is small, but this is a rare condition and the results should bolster further development in this important area.  

 Methods: Pindel and grids are superior methods for finding SV's than MANTA, but the difference would be minor in this type of study. Other methods are acceptable. Since this is a small study, the definition of differential expression is between the two different conditions, this is a limitation of the study - more accurate assessment of expression would be a non-parametric ranking of gene expression- how unlikely either very high or very low expression is across a cohort of similar tumors. This would be a meta-analysis, likely using public data in cBioPortal or data from TGen or USC biobanks, and while useful would likely exceed the scope of the present project. This limitation is also acceptable. This reviewer would like a separate section or note near the methods section describing the statistical limitations of the cohort, the study, and the statistical design. While mentioned in the discussion (small sample size, non-coding RNA's are assigned slightly more importance than may be warranted) this reviewer suggests the methods section should note that this study is not a meta-analysis, the differential expression is highly limited, non-parametric analyses have limited utility, and the pathway analysis and other interpretations should be presented in the context of the study limitations, rather than a sentence in the discussion. Minor additions to the data addressing these limitations would provide readers a way to understand that extending the results to a biological cause or even to a pathway is pure speculation. I would much prefer the authors use phrases like "evidence suggests genes X, Y, Z that were found through our analysis are implicated in the functions shown in our genetic pathway figures." The way it's written, there is more confidence in the interpretation of the biological role of the detected gene expression than may be warranted. It would be beneficial to highlight similar findings in published journals concerning similar tumor types, similar benign vs. metastatic cases, and perhaps making a case for a set of commonly activated genes or types of genes that appear in published/public studies of metastasis/benign tumors.

 Overall the study brings up good points, the NGS analysis is fine, the WTS and WES are an important combination, and releasing these data into the public domain will spur further studies from these important data. The biological analysis is extensive but may be a little overstated. The conclusions are warranted. The text and writing style are acceptable, the references are well-chosen but could be bolstered by finding any studies between met/benign tumors in similar tissues that might have found similar over/underexpressed genes.

 Recommend publish with minor revisions.

Is the work clearly and accurately presented and does it cite the current literature?

Yes

If applicable, is the statistical analysis and its interpretation appropriate?

Yes

Are all the source data underlying the results available to ensure full reproducibility?

Yes

Is the study design appropriate and is the work technically sound?

Yes

Are the conclusions drawn adequately supported by the results?

Yes

Are sufficient details of methods and analysis provided to allow replication by others?

Yes

Reviewer Expertise:

WTS/WES NGS bioinformatics, cancer biology, immunology.

I confirm that I have read this submission and believe that I have an appropriate level of expertise to confirm that it is of an acceptable scientific standard, however I have significant reservations, as outlined above.

# Response

**Published:** 2023-10-04

**Authors:** Mackenzie Postel

**Affiliations:** USC Norris Comprehensive Cancer Center, USA

**REVIEWER** : “This reviewer would like a separate section or note near the methods section describing the statistical limitations of the cohort, the study, and the statistical design. While mentioned in the discussion (small sample size, non-coding RNA's are assigned slightly more importance than may be warranted) this reviewer suggests the methods section should note that this study is not a meta-analysis, the differential expression is highly limited, non-parametric analyses have limited utility, and the pathway analysis and other interpretations should be presented in the context of the study limitations, rather than a sentence in the discussion… I would much prefer the authors use phrases like "evidence suggests genes X, Y, Z that were found through our analysis are implicated in the functions shown in our genetic pathway figures." 

**AUTHOR RESPONSE** : Thank you for your feedback. In our updated manuscript, we have added an explicit “limitations” section, as well as specified the parametric and semi-parametric differential expression packages utilized by the iDEP tool. We have also endeavored to qualify results such that readers interpret them within the context of study limitations.

 Note: we also cited 11 new papers, related to reviewers’ suggestions, 3 of which were published after we had written our original manuscript.
